# Bilateral optic disc oedema and associated optic neuropathy in the setting of FOLFOX chemotherapy

**DOI:** 10.1186/1471-2415-13-42

**Published:** 2013-08-08

**Authors:** Liam D Turner, John D Harrison

**Affiliations:** 1 Royal Brisbane and Women’s Hospital, Brisbane, QLD 4006, Australia; 2 Princess Alexandra Hospital, Brisbane, QLD 4102, Australia

**Keywords:** Optic neuropathy, Ischaemic optic neuropathy, FOLFOX, Fluorouracil

## Abstract

**Background:**

To report a case of bilateral optic disc oedema and associated optic neuropathy in the setting of FOLFOX chemotherapy.

**Case presentation:**

A case of a 57-year-old male being treated with FOLFOX chemotherapy for stage 3B colorectal cancer, who developed bilateral optic disc oedema and associated left sided optic neuropathy is described. The patient presented following cycles 7, 8 and 9 of chemotherapy with a history of bilateral simultaneous intermittent inferior altitudinal field defects. These episodes progressed to bilateral optic nerve oedema and a subsequent left sided optic neuropathy. The patient’s symptoms and oedema regressed with discontinuation of chemotherapy.

**Conclusion:**

This is the first report suggesting a vasospastic role of 5-fluoruracil in 5-FU associated optic neuropathy. It highlights that 5-FU may have the potential to cause arterial vasospasm outside the cardiac vasculature, resulting in end-organ optic nerve ischaemia.

## Background

The following case report highlights the presence of bilateral optic disc oedema with associated optic neuropathy whilst undergoing FOLFOX chemotherapy. FOLFOX chemotherapy consists of oxaliplatin, fluorouracil and leucovorin and has been used for the treatment of stage three colorectal cancer since the release of early data from the phase III MOSAIC trial in 2003 [1]. 5-Fluorouracil (5-FU) is an antimetabolite, which inhibits the action of thymidylate synthase and ultimately interferes with DNA replication [1]. 5-FU has been shown to have significant arterial vasospastic effects, which involves predominantly the coronary arteries, resulting in ischaemia and subsequent infarction of the myocardium, arrhythmias and sudden cardiac death [2-4]. Optic neuropathy has been documented previously when patients have been undergoing 5-FU treatment, with four identified cases of optic disc oedema and associated optic neuropathy in the setting of continuous 5-FU infusion [5-8]. Furthermore, the National Registry lists optic neuropathy as “possibly linked” to 5-FU treatment, highlighting several cases in the academic literature and within the registry [9]. The mechanism for optic neuropathy has not been documented, with some authors only postulating that 5-FU has a toxic effect on the optic nerve, which potentially may occur as a result of dihydropyrimidine dehydrogenase deficiency (DPD), which is the rate-limiting step in the metabolism of 5-FU [5]. Following is a case study where the patient developed optic disc oedema and associated optic neuropathy whilst undergoing 5-FU treatment, which further provides evidence for a possible association between 5-FU and optic neuropathy, providing the opportunity to discuss potential mechanisms for the associated toxicity.

## Case presentation

A 57-year-old male with stage 3B colorectal cancer was referred by Medical Oncology to the Royal Brisbane Hospital Ophthalmology department, with simultaneous transient bilateral inferior altitudinal defects that would last up to 10 seconds in duration. These defects had commenced approximately four weeks earlier. The patient had no atherosclerotic risk factors (e.g. smoking, hypercholesterolaemia, hypertension, diabetes), or suffer from episodes of systemic hypotension, or obstructive sleep apnoea. On initial presentation to the Ophthalmology department, the patient had just completed cycle 8 of an intended 12 cycles of FOLFOX adjuvant chemotherapy with curative intent. The patient’s dosing schedule was oxaliplatin 165 mg (Day 1), fluouracil 780 mg bolus dose (Day 1) with a subsequent infusion of 1170 mg over 48 hours, and leucovorin 390 mg (Day 1 + 2). The patient’s symptoms had occurred during cycle 7 within the continuous infusion period, and for a few days following the completion of the infusion. On initial examination the patient’s visual acuity was 6/5 bilaterally. Ophthalmic examination, computerized perimetry and OCT retinal nerve fiber layers were unremarkable, with no evidence of a disc at risk at this time. The bilateral simultaneous inferior altitudinal field defects subsequently recurred with cycles 8 and 9 of chemotherapy during the continuous infusion and for a few days following the completion of the continuous infusion, but had not occurred in the periods between cycles.

**Figure 1:**
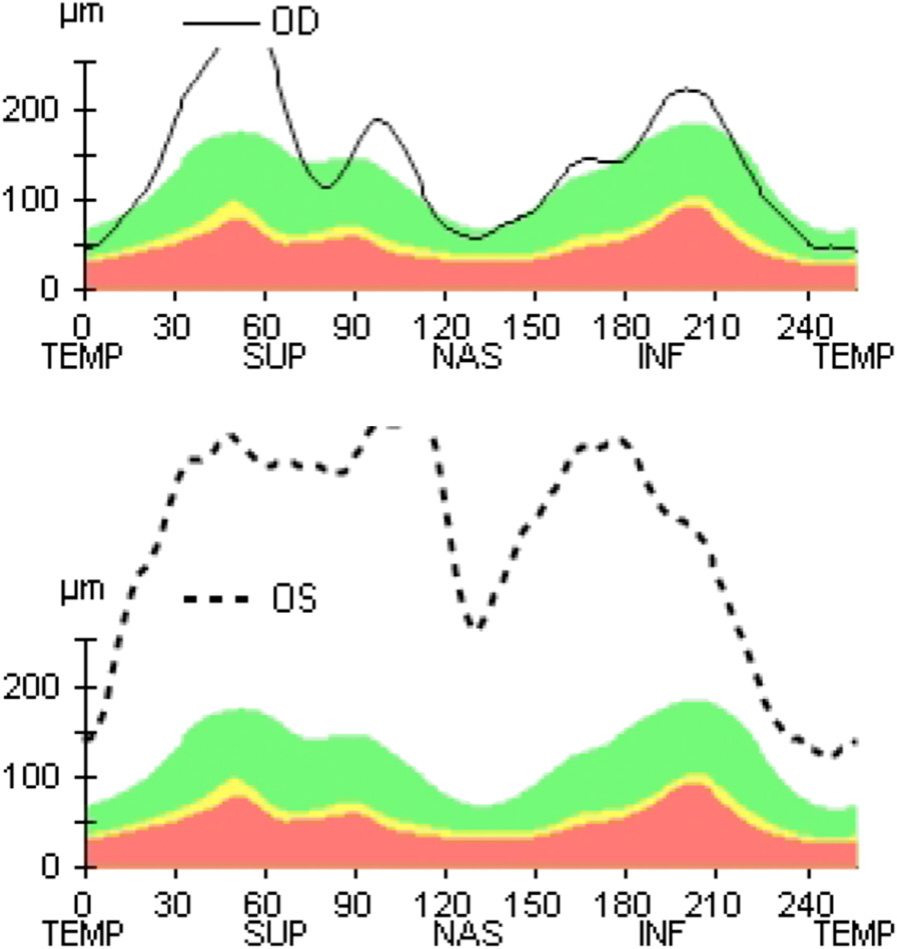
(OCT RNFL Optic Discs).

**Figure 2:**
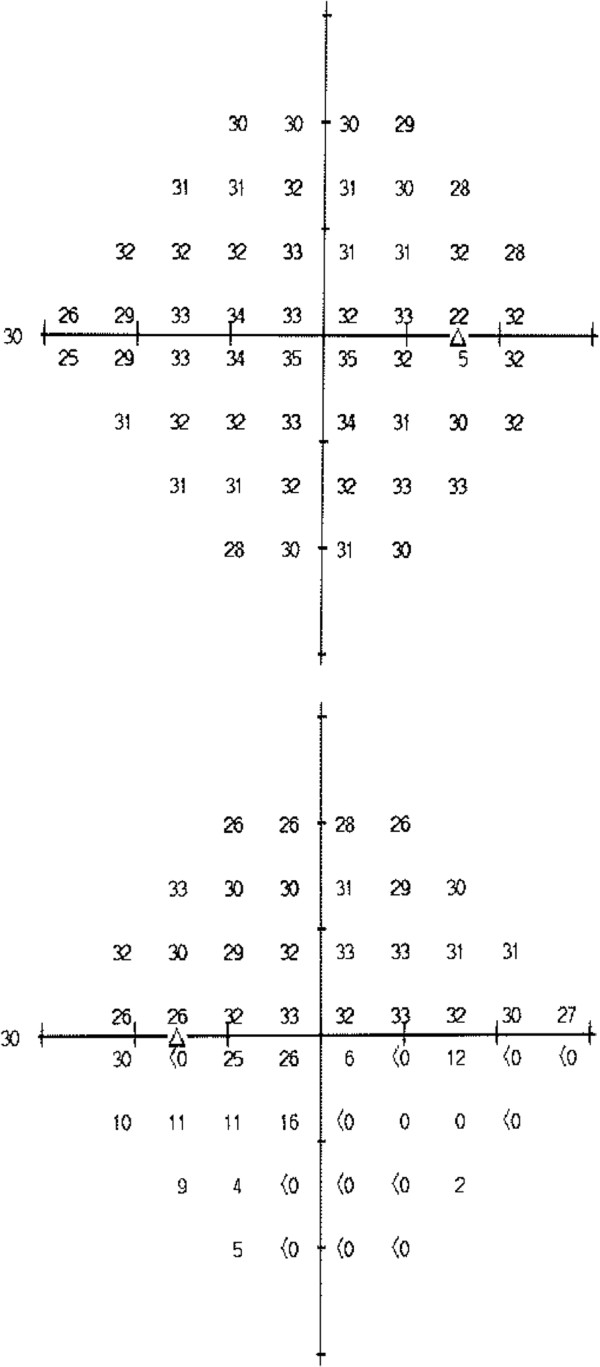
(CP 24-2).

The patient represented two weeks later, upon completion of cycle 9 of chemotherapy, with a persistent complete left inferior altitudinal field defect. Ophthalmic examination at this time revealed visual acuities of right 6/5-1, left 6/7.5 with a left relative afferent pupillary defect. Dilated fundal examination revealed diffuse left sided optic disc swelling and swelling of the superior quadrant of the right disc (Figure 1: OCT RNFL Optic Discs). The patient subsequently went on to have formalized fields, which confirmed the left inferior altitudinal defect and no obvious field defect on the right (Figure 2: CP 24-2). At this stage, the patient’s chemotherapy was ceased after careful discussion with the medical oncology team, who in their expert opinion felt that the threat of further visual loss was greater than the potential benefit to be gained from further chemotherapy in reducing recurrence of the disease.

Investigations were subsequently undertaken to rule out secondary causes of the optic disc oedema (i.e. toxic, infiltrative). The patient underwent blood examination (e.g. CRP, ESR, FBC, Paraneoplastic Panel Screen), duplex carotid ultrasound, holter monitor, MRI brain and orbits and lumbar puncture. All investigations were inconclusive. The patient was prescribed low dose aspirin 100 mg/day and brimonidine tartate 2 times/day. Over the course of approximately three months the patient’s symptoms did not progress without the chemotherapy treatment and the patient ceased to experience any further episodes of inferior altitudinal field defects on the right side (the left side now had a persistent defect). Serial ophthalmic examination revealed a stable visual acuity with resolution of optic disc oedema and the gradual appearance of a pale superior left optic nerve. Repeat fields showed a stable left inferior altitudinal defect with no progression.

## Conclusions

We propose that the episodes of bilateral simultaneous altitudinal field defects and resultant bilateral optic disc oedema with associated left optic neuropathy may be the result of arterial vasospasm induced by 5-FU in the short posterior ciliary arteries. 5-FU has been proven to initiate arterial vasospasm, with both animal and human studies that have demonstrated a dose response relationship that abates with cessation of drug administration [4,10]. Imaging studies have demonstrated that this vasospasm is not specific to the coronary vasculature and has been shown to be present within peripheral arteries. Ultrasound evidence exists that demonstrates the occurrence of arterial vasospasm in the brachial arteries following administration of 5-FU [11,12]. Furthermore, 5-FU has been shown in vitro to induce vasoconstriction of vascular smooth muscle cells via activation of protein C, which resolves with administration of protein kinase inhibitors [4]. This highlights a biological plausibility to our case. Moreover, the episodes of inferior altitudinal field defects occurred in conjunction with the 5-FU infusion, reflecting a close temporal relationship between drug delivery and the development of symptoms that also disappeared with cessation of the infusion. These symptoms of altitudinal field defects recurred with subsequent cycles of reinfusion ultimately resulting in sufficient ischaemia to produce infarction in the form of a non-arteritic anterior ischaemic optic neuropathy (NAION). Upon cessation of the chemotherapy the symptoms of intermittent altitudinal field defects (in the right eye) settled and there was no progression of the ischaemic optic neuropathy (in the left eye).

We believe that given the absence of: (1) atherosclerotic risk factors; (2) a holter monitor showing sinus rhythm; and (3) duplex carotid ultrasound revealing no evidence of atherosclerotic disease, that an embolic source is unlikely. In addition, a vasculitic cause (i.e. Giant Cell Arteritis) is also less plausible because of a normal ESR, CRP, no history consistent with temporal arteritis, and failure to progress despite the absence of corticosteroid treatment. It is also considered highly improbable that individual emboli could be disseminated to both short posterior ciliary arteries on the right and left eye, at exactly the same time on multiple occasions over a period of three cycles of chemotherapy. It is not possible to prove from a single case that 5-FU causes short posterior ciliary artery vasospasm and ultimately NAION. However, given the biological plausibility of the mechanism and the close temporal association, we are of the opinion this should at least be considered. Delval and Klastersky [5] discount an ischaemic or vascular cause to 5-FU’s mechanism of optic neuropathy, based on the absence of an altitudinal field defect, flame-shaped haemorrhages of the disc, history of diabetes or hypertension, or evidence for cardioembolic disease in their patient. Given the absence of these risk factors (i.e. atherosclerotic, cardioembolic), the vasospastic properties of 5-FU should be considered as the primary mechanism for the apparent vascular related visual field defects experienced by our patient. Furthermore, whilst oxaliplatin is known to cause peripheral neuropathy, and reports are present in previous literature for its potential association with optic neuropathy [13,14], the vascular nature of our patients visual obscurations are not consistent with a primary axonal mechanism as postulated for with oxaliplatin. Finally, leucovorin is given to potentiate the effects of 5-FU in FOLFOX chemotherapy.

5-FU has been reported as a possible cause of optic neuropathy in the past [5-8]. It is proposed that the transient visual disturbance experienced with 5-FU infusion treatment is in fact a result of short-posterior ciliary artery vasospasm, which further results in transient episodes of ischaemia to the optic nerve. These patients may never come to the attention of ophthalmologists as the disturbances are generally thought of as transient, with no long lasting effects. Our case may represent an individual who experienced these transient visual disturbances and whose arterial vasospasm may have been more severe than usually encountered to result in permanent ischaemia and necrotic tissue damage to the optic nerve head. We believe that all patients undertaking FOLFOX chemotherapy who experience visual disturbances should be examined by an ophthalmologist to determine the exact nature of these disturbances and to further investigate and interpret evidence to identify a potential cause.

## Requesting consent statement

Written informed consent was obtained from the patient for publication of this case report and any accompanying images. A copy of the written consent is available for review by the Editor of this journal.

## Competing interests

The authors declare that they have no competing interests.

## Authors’ contributions

LT initially identified the case and after discussion with JH decided to write up the case. A literature review was conducted by LT and subsequently a manuscript was formulated by LT and JH. Both parties approved the final manuscript.

## Pre-publication history

The pre-publication history for this paper can be accessed here:

http://www.biomedcentral.com/1471-2415/13/42/prepub

## References

[B1] Andre TBC Navarro M Improved overal survival with oxaliplatin, fluoruracil, and leucovorin as adjuvant treatment in stage II or III colon cancer in the MOSAIC trial J Clin Oncol 2009 27 3109 3116 10.1200/JCO.2008.20.6771 19451431

[B2] Sorrentino MF Truesdell AG 5-fluouracil-induced coronary thrombosis J Cardiol Cases 2012 6 e20 e22 10.1016/j.jccase.2012.03.011 PMC6269271 30532939

[B3] Kosmas C Kallistratos M Kopterides P Cardiotoxicity of fluoropyrimidines in different schedules of administration: A prospective study J Cancer Res Clin Oncol 2008 134 75 82 17636329 10.1007/s00432-007-0250-9 PMC12161746

[B4] Mosseri M Fingert H Varticovski L Chokshi S Isner J vitro evidence that myocardial ischaemia resulting from 5-fluouracil chemotherapy is due to protein kinase c-mediated vasoconstriction of the vascular smooth muscle Cancer Res 1993 53 3028 3033 8391384

[B5] Delval L Klastersky Optic neuropathy in cancer patients. Report of a case possibly related to 5 fluorouracil toxicity and review of the literature J Neurooncol 2002 60 165 169 10.1023/A:1020613600826 12635664

[B6] Adams J Bofenkamp TM Kobrin J Wirtschafter JD Zeese JA Recurrent acute toxic optic neuropathy secondary to 5-FU Cancer Treatment Reports 1984 68 565 566 6704988

[B7] Langley J Rosato FE El-Mahdi A Primary malignant hemangioendothelioma of the liver: survival following nonoperative treatment J Surg Oncol 1978 10 533 541 10.1002/jso.2930100610 732337

[B8] Weiss A Jackson LG Carabisi R An evaluation of 5-fluorouracil in malignant disease Ann Intern Med 1961 55 731 741 10.7326/0003-4819-55-5-731 14005852

[B9] Fraunfelder F Fraunfelder FW Chamber WA Clinical 2008 Saunders Elsevier: Ocular Toxicity

[B10] Labianca R Beretta G Clerici M Frascini P Luporini G Cardiac toxicity of 5-fluouracil: A study of 1083 patients Tumori 1982 68 505 510 7168016 10.1177/030089168206800609

[B11] Luwaert R Descamps O Majois F Chaudron J Beauduin M Coronary artery spasm induced by 5-fluouracil Eur Hear J 1991 12 468 470 10.1093/oxfordjournals.eurheartj.a059919 2040332

[B12] Sudhoff T Enderle M Pahlke M 5-fluouracil induces arterial vasoconstriction Ann Oncol 2004 15 661 664 10.1093/annonc/mdh150 15033676

[B13] Mesquida M Sanchez-Dalmau B Ortiz-Perez S Pelegrin L Molina-Fernandez JJ Figueras-Roca M Casaroli-Marano R Adan A Oxaliplatin-related ocular toxicity Case Reports Oncol 2010 3 423 427 10.1159/000322675 PMC2999736 21151636

[B14] Park S Lin CS Krishnan AV Goldstein D Friedlander ML Kiernan MC Oxaliplatin-induced neurotoxicity: changes in axonal excitability precede development of neuropathy Brain 2009 132 2712 2723 10.1093/brain/awp219 19745023

